# Effect of wearing a helmet on the occurrence of head injuries in motorcycle riders in Benin: a case-control study

**DOI:** 10.1186/s40621-021-00311-3

**Published:** 2021-05-10

**Authors:** Bella Hounkpe Dos Santos, Yolaine Glele Ahanhanzo, Alphonse Kpozehouen, Donatien Daddah, Emmanuel Lagarde, Yves Coppieters

**Affiliations:** 1 Ecole de Santé Publique, Université Libre de Bruxelles, Bruxelles, Belgium; 2 Institut Régional de Santé Publique, PB 384, Ouidah, Bénin; 3 Université de Bordeaux, Bordeaux, France

**Keywords:** Head injuries, Motorcycles, Helmet use, Road crash, Benin

## Abstract

**Background:**

In Benin, motorcycles are the main means of transport for road users and are involved in more than half of crashes. This study aims to determine the effect of wearing a helmet on reducing head injuries in road crashes in Benin.

**Methods:**

This case-control study took place in 2020 and focused on road trauma victims. The sample, consisting of 242 cases (trauma victims with head injuries) for 484 controls (without head injuries), was drawn from a database of traffic crash victims recruited from five hospitals across the country from July 2019 to January 2020. Four groups of independent variables were studied: socio-demographic and economic variables, history, behavioural variables including helmet use and road-related and environmental variables. To assess the shape of the association between the independent variables and the dependent variable, a descending step-by-step binary logistic regression model was performed using an explanatory approach.

**Results:**

Fewer of the subjects with a head injury were wearing a helmet at the time of the crash 69.8% (95% CI = 63.6–75.6) compared to those without a head injury 90.3% (95% CI = 87.3–92.8). Adjusting for the other variables, subjects not wearing helmets were at greater risk of head injuries (OR = 3.8, 95% CI (2.5–5.7)); the head injury rating was 1.9 (95% CI = 1.2–3.3) times higher in subjects who were fatigued during the crash than among those who were not and 2.0 (95% CI = 1.2–3.3) times higher in subjects with no medical history.

**Conclusion:**

Failure to wear a helmet exposes motorcyclists to the risk of head injuries during crashes. It is important to increase awareness and better target such initiatives at the subjects most at risk.

## Background

Road traffic crashes have a heavy burden of disease and mortality around the world. These crashes are the leading cause of injury and are the eighth leading cause of death worldwide (WHO 2018). Each year, they are responsible for 1.25 million deaths. The World Health Organization (WHO) observed the highest mortality rates in the African region, with 26.6 deaths per 100,000 inhabitants in this region against 17.4 deaths per 100,000 inhabitants worldwide (WHO 2018). Benin is a country in West Africa with an estimated population of around 11,857,627 in 2019 (MS 2020). In Benin, each year, from 2010 to 2016, the police recorded an average of 6000 crashes and 700 deaths (CNSR 2017). In 2019, trauma was the fourth cause of health care utilization and road crashes accounted for one third of all injuries reported by the health system (MS 2020).

Those most exposed to road crashes, serious injuries and deaths from road crashes are vulnerable users such as pedestrians, cyclists and riders of motorised two-wheelers and their passengers (WHO 2018; Lin and Kraus 2009; Tin Tin et al. 2010; Bouaoun et al. 2015; Naci et al. 2009). In African region, 7 to 16% of road deaths are among motorcyclists (WHO 2018; Naci et al. 2009). The increase in the number of motorcycles and motorcycle trips in the African region is one of the factors contributing to the growth of road crashes (WHO 2018; Naci et al. 2009). Benin is characterized by the strong predominance of two-wheeled motorcycles as a means of moving populations and more than 55% of households own motorcycles (Pochet et al. 2017). In Benin, two-wheelers were involved in around 50% of the roads crashes and 50% of road deaths are among these road users (CNSR 2017). Among the motorcyclists, although limb trauma is the most common injury in traffic crashes, head injuries are more serious. They are responsible for around half of the deaths (Lin and Kraus 2009).

The main causes of these crashes are speeding, driving under the influence of alcohol or any psychoactive substance, the lack of a helmet, seat belt, or safety devices for children, and distracted driving due to mobile phones (WHO 2018). In addition to these behavioural factors, there are those related to the condition of the roads or the condition of the vehicles (WHO 2018). Some authors have noted several factors that can influence the attitudes and behaviours of drivers, such as driver inexperience, driving long hours in a day, working late hours, the territorial context in which the drivers live, driver training, compliance with laws on lighting and visibility, and possession of an individual driver’s license, up-to-date parts and motorcycle insurance (Tumwesigye et al. 2016; de Oña et al. 2014a; de Oña et al. 2014b; Borowsky et al. 2010; Staton et al. 2016). The WHO recommends the use of helmets as one of the main means of preventing road traffic injuries (WHO 2018; OMS 2011). Several authors have confirmed the importance of helmet use in reducing head injuries, injury severity and fatalities among motorcyclists (Lin and Kraus 2009; French et al. 2009; Brown et al. 2011; Singleton 2017; Ankarath et al. 2002; Liu et al. 2008). According to the authors, in addition to helmet use, others factors could influence the occurrence of head, maxillofacial in cyclists or motorcyclists victims of traffic injuries. The factors most found in the literature were: collision object (Orsi et al. 2014; da Nóbrega et al. 2017; Lin et al. 2003), suburban and rural zones (da Nóbrega et al. 2017; Lin et al. 2003; Baru et al. 2019), crashes occurring at dawn or night hours (Lin et al. 2003; Baru et al. 2019; Lam et al. 2020), driving under the influence of alcohol (Baru et al. 2019; Lam et al. 2020), using mobile phones and riding at the same time (Lam et al. 2020), speeding (Lin et al. 2003), types of crashes and carrying heavier loads (Oginni et al. 2009).

In Benin, a Decree of April 1972 established the compulsory wearing of helmets by drivers and passengers of two-wheeled vehicles or the like. For decades, this law was not enforced. Since 2014, several actions have been carried out to ensure its effective implementation by motorcycle drivers in large cities. There are no data on the proportion of motorcyclists wearing helmets in Benin. The few data available come from hospital studies. Before the implementation of the helmet law, 96% of motorcyclists admitted to the National Hospital-University Centre of Cotonou for cranio-encephalic trauma caused by road crashes did not wear a helmet (Tidjani et al. 2018). One year later, after increased checks on the wearing of helmets, there was a reduction in the frequency of head injuries, but also an increase in the proportion of trauma victims wearing helmets (Hode et al. 2017). It is relevant, after several years of application of this law on the wearing of helmets in Benin, to verify whether the subjects wearing helmets are less at risk of head injuries, and to identify the other factors likely to influence the occurrence of those head injuries. The objective of this study was to determine the effect of wearing a helmet on the occurrence of head injuries in road crashes in Benin.

## Materials and methods

### Type of study

This is a case-control study that took place in 2020.

### Study population, inclusion and exclusion criteria

The target population consisted of motorised two-wheeled vehicle drivers who were victims of road traffic injuries. The cases included traumatized persons with head injuries with or without injuries to other locations. Controls were those with no head injuries but only at other locations. Subjects for whom information related head injuries and other variables (helmet wearing, etc.) was not provided and those who did not give their consent were not included in the study (*n* = 175).

### Data source and selection of participants

**Fig. 1:**
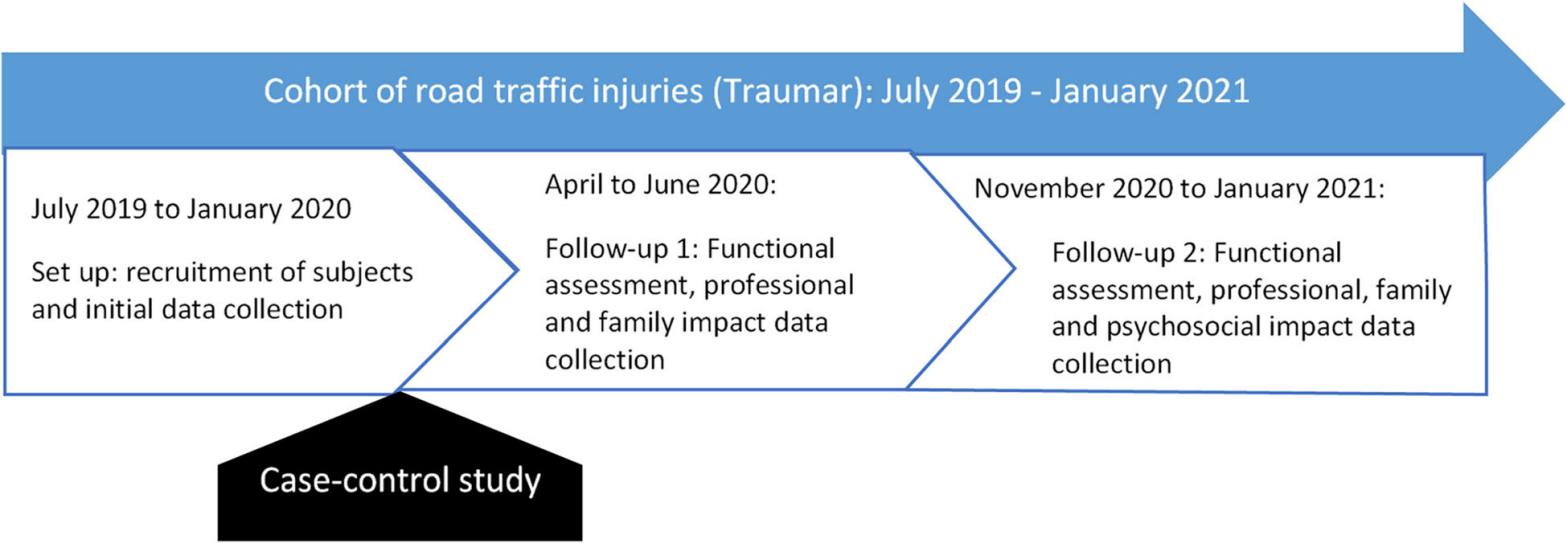
Positioning of the case-control study in the TraumAR

The present study was carried out on the cohort of road traffic injuries called TraumAR (see Fig. 1), set up by a team of researchers with the support of the Multidisciplinary Research Project for the Prevention of Road Accidents (ReMPARt). It was formed through the recruitment of subjects in two hospitals in the north of Benin (Boko district hospital and the Regional Teaching Hospital of Borgou in Parakou) and three in the south (Menontin district hospital, the National Teaching Hospital Hubert Koutoukou Maga of Cotonou, the Regional Teaching Hospital of Ouémé in Porto-Novo). These hospitals were selected on the basis of statistics for trauma caused by road crashes, but also in order to have hospitals in the south and north of the country. In these hospitals, the subjects admitted as emergency patients for road crash trauma were recruited from 01 July 2019 to 31 January 2020 to form the Traumar. For the implementation of this cohort, some research associates were recruited and trained to complete the collection tool installed on tablets using Kobocollect. They were in the hospitals and worked with the teams of health workers. They judged the appropriate time to approach trauma victims and/or their caregivers, usually after the on-call staff had met victims at the hospital and given them first aid. After obtaining their free and informed written consent, each patient submitted to a questionnaire to prospectively collect the exhaustive data needed to create the Traumar database. These data collected from the subjects were supplemented by other data obtained from the medical record and from health workers. Data obtained from medical records were related to clinical and paraclinical examination, diagnosis and care administered, as well as evolution. All the data recorded, which made it possible to establish the TraumAR database, related to general information, crash risk factors, severity factors, clinical, paraclinical, therapeutic and monitoring information, and patient outcome.

**Fig. 2:**
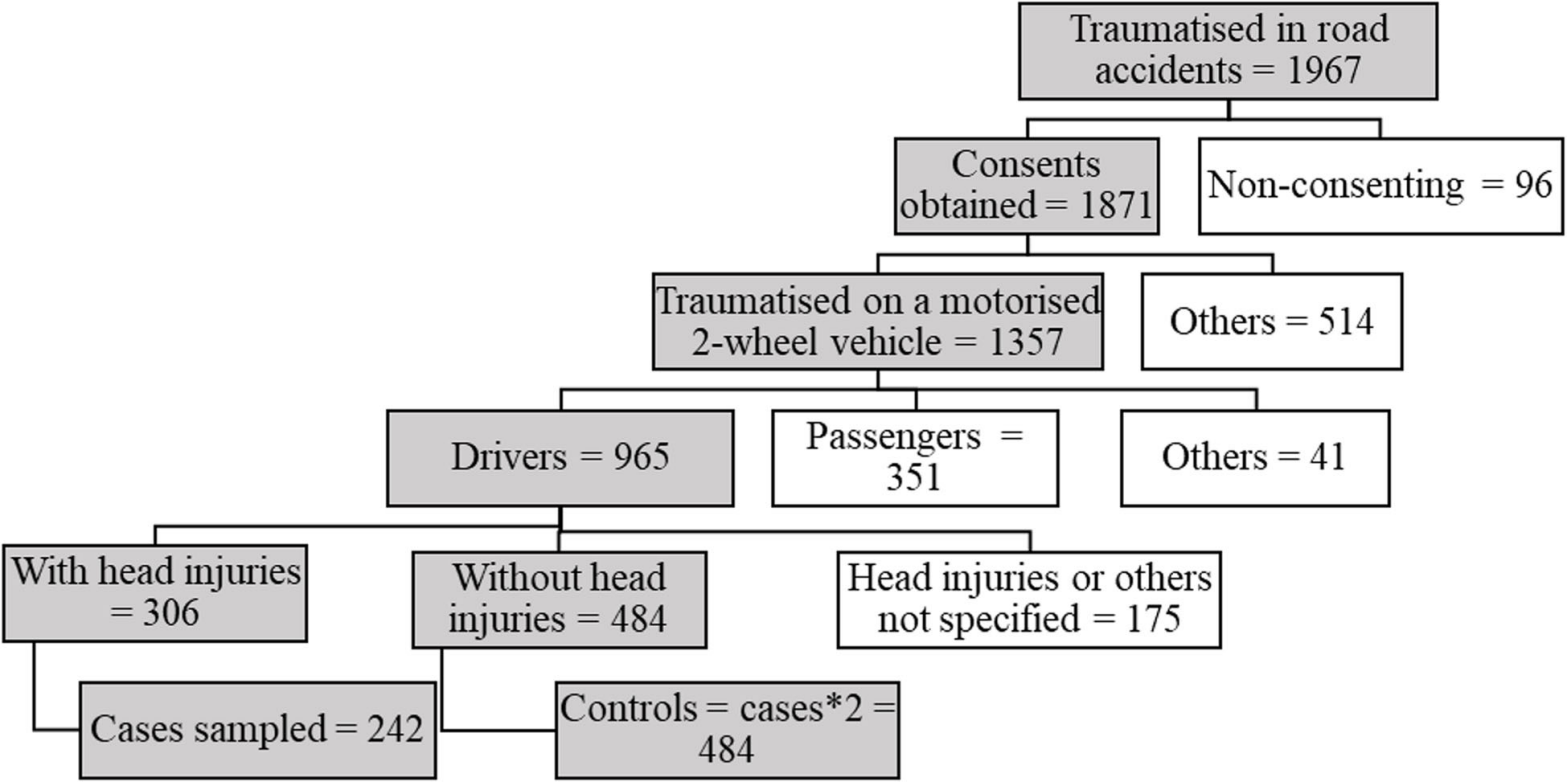
Selection process for cases and controls in the TraumAR database (non-eligible records have a white background)

The subjects were divided into two groups: the group of cases presenting with head injuries (306 individuals) and the group of controls without head injuries (484). In order for the number of cases to correspond to half of the total number of controls, a simple random selection without replacement of 242 cases was carried out using the “sample” command in Stata (Fig. 2).

### Sample size

In order to define the number of subjects necessary for our study, we used the formula developed by Machin et al. (Machin et al. 2018). To do this we considered one (WHO 2018) case for two (MS 2020) controls (φ = 2), a power of 80% (1-β), a confidence level of 95% (α = 5%), a minimum difference in odds ratio expected between the groups OR_plan_ of 1.8. In the absence of previous research on motorcycle riders without experiencing head injuries while wearing a helmet, we took 50% as a probability of exposure in controls π_2_.

A minimum sample size of 142 cases and 284 controls was required. We were able to include 242 cases and 484 controls in the study.

### Variables

To perform this study, the binary dependent variable was “head injuries”. Head injuries were defined as any post-traumatic injury to the head observed on clinical examination in a conscious or unconscious road accident trauma patient. The diagnosis was made by a general practitioner or specialist, confirmed or not by paraclinical examinations (imaging) such as radiography. Four groups of factors likely to explain the occurrence of head injuries in motorcyclists who experienced trauma were identified, as independent variables, following a review of the literature (WHO 2018; Lin and Kraus 2009; French et al. 2009; Brown et al. 2011; Singleton 2017; Ankarath et al. 2002; Liu et al. 2008; Orsi et al. 2014; da Nóbrega et al. 2017; Lin et al. 2003; Baru et al. 2019; Lam et al. 2020; Oginni et al. 2009). The socio-demographic and economic factors were age, sex, body mass index (BMI), which is weight in kilograms divided by height in square meters, ethnicity, professional situation, marital status, household size and the number of dependent children aged 0–18. Another group of factors was the history, such as medical history (chronic diseases such as high blood pressure and other cardiovascular pathologies, diabetes, asthma, vision problems, sleep disorders, epilepsy, etc.), history of traffic crashes, driving experience, and use of drugs, stimulants, alcohol or tobacco. The road and environmental factors used were the type of road, the condition of the road, antagonist in the crash, the weather conditions, the time of day, and the level of visibility (overall visibility in the environment, appreciated by the patient). Behavioural factors were helmet use, telephone use, distraction and fatigue/drowsiness at the time of the crash. The main exposure sought was the wearing of helmets.

### Data processing and analysis

Stata 15.1 was used for data processing and analysis. Variables were described for cases and controls. The quantitative variables were expressed as a mean followed by their standard deviation because their distributions, verified graphically (histogram, box-plot), were normal. The qualitative variables were described by their frequencies. The dependent variable, head injuries was cross-tabulated with each of the independent variables. Chi-square statistical test was used for comparison of proportions when conditions were true. Student’s test was used for the comparison of continuous variables. For this test, equality of variances was tested using Levene’s robust test. If this test was significant, the Hartley test was performed. A logistic regression was also performed in a univariate analysis. For this regression, the indicator to measure the association was the odds ratio (OR) followed by its 95% confidence interval (95% CI).

Modelling was done to assess the shape of the association between the independent variables and the dependent variable using a binary logistic regression. The option chosen was a top-down step-by-step, explanatory model. The variables entered in the initial multi-variate model were those with a *p*-value ≤0.1 on univariate analysis. The variables were gradually removed from the initial model taking into account their *p*-value (greater than 0.05). The final model was the one in which all the variables were significant. In the final model, collinearity between the variables was sought. The residuals (Pearson, standardised and deviance) were calculated to identify influencing values and outliers. The model’s goodness of fit was checked with the Hosmer-Lemeshow test as well as its specification (linktest). The model was adequate for a *p*-value > 0.05. The significance level retained was 5% for all the tests.

## Results

### Comparison of included to excluded subjects

Of the 965 motorcycle drivers, 175 were excluded due to missing data. Subjects excluded from the study were compared to those included on the basis of gender, the only variable with no missing data among the excluded. Female subjects represented 10% of all included subjects (*n* = 726) and 11% of those excluded (*n* = 175). There was no significant difference between the two groups.

### Sociodemographic and economic characteristics of the subjects

**Fig. 3:**
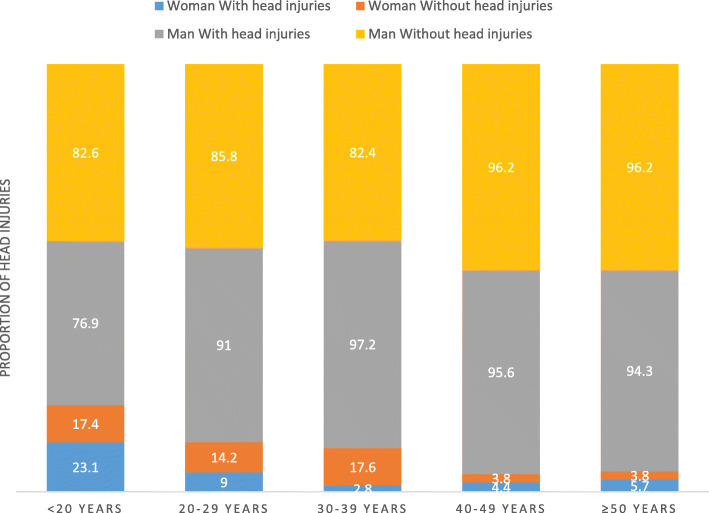
Distribution of cases and controls according to their sex and age

Apart from gender, the socio-demographic characteristics (Table 1) of motorcycles riders traumatised in road crashes who experienced a head injury (cases) were not different from those of riders who did not experience a head injury (controls). The mean age of the subjects did not differ in the two groups (35.6 ± 12.7 years for those with head injuries versus 36.7 ± 12.1 years for those without head injuries). The majority subjects were male in both groups: 93.4% in the group with head injuries versus 88.6% in the subjects without head injuries. Head injuries occurred significantly more in men than in women (*p* < 0.05). However, the difference in the occurrence of head injuries between men and women is most noticeable in the 30 to 39 year age group (Fig. 3). The Fon or related ethnic group (64.0% in the cases and 70.4% in the controls) was the most represented. The majority (about 83%) of the cases and controls were in employment. Married subjects were more numerous in both groups. The average household size was around five people and the number of dependent children under 18 was on average two in both groups. The mean BMI was normal in both groups at around 23 kg/m^2^.

**Table 1:** Distribution of cases and controls according to socio-demographic and economic factors

*Variables*	Head injuries (% or Mean ± sd)		*p*-value
	Yes (*n* = 242)	No (*n* = 484)	
Sex			0.042
*Female*	6.6	11.4	
*Male*	93.4	88.6	
Ethnic group			0.102
*Bariba*	8.3	6.2	
*Dendi*	2.5	3.3	
*Fon and related*	64.0	70.4	
*Peulh*	7.0	2.9	
*Nago and related*	11.6	12.2	
*Other ethnicities*	6.6	5.0	
Professional situation			0.334
*Unemployed*	1.6	3.9	
*In employment*	83.1	83.3	
*Training*	15.3	12.8	
Employment sector			0.630
*Public or denominational*	12.5	11.2	
*Private*	87.5	88.8	
Marital status			0.932
*Single*	30.6	29.3	
*Married or engaged*	67.3	68.4	
*Divorced or widowed*	2.1	2.3	
BMI	23.4 (3.8)	23.7 (3.5)	0.308
Age	35.6 (12.7)	36.7 (12.1)	0.262
Household size	5.0 (2.6)	4.9 (2.8)	0.620
Number of children < 18 years	2.0 (1.7)	1.9 (1.6)	0.279

### Attitudes and behaviours of motorcycle riders

The proportion of the study subjects wearing helmets was higher in the group without head injuries (90.3% vs. 69.8%; *p* = 0.000) (Table 2). More subjects who had a head injury (cases) drove in a state of fatigue or drowsiness than the controls (16.5% vs 9.3%; *p* = 0.004). In both cases and controls, very few subjects regularly adopted certain risky behaviours such as distracted driving or using the telephone while driving. Of the subjects who had head injuries, 3.3% were using their phones at the time of the crash compared to only 0.8% of those who did not have head injuries (*p* = 0.01). In contrast, there was not a significant difference between the two groups with regard to distracted driving (15.7% of those with head injuries versus 13.8% of those without head injuries) (Table 2).

**Table 2:** Distribution of cases and controls according to behavioural factors

*Variables*	Head injuries (% or Mean ± sd)		*p*-value
	Yes (*n* = 242)	No (*n* = 484)	
Helmet use			0.000
*Yes*	69.8	90.3	
*No*	30.2	9.7	
Distracted during the crash			0.502
*Yes*	15.7	13.8	
*No*	84.3	86.2	
Telephone use during the crash			0.014
*Yes*	3.3	0.8	
*No*	96.7	99.2	
Fatigue/drowsiness during the crash			0.004
*Yes*	16.5	9.3	
*No*	83.5	90.7	

### Road and environmental factors

Almost half of the cases and controls’ crashes occurred during the day (45.9% for cases compared to 54.5% for controls). Other antagonist in the crash was more often a moving vehicle, both for the subjects with head injuries (64.9%) and those without (77.1%). However, the cases had more crashes where there was no antagonist compared to the controls (*p* = 0.017). Overall, visibility during the crashes was good among cases (64.5%) as well as among controls (76.2%). The subjects who had head injuries had more crashes when the level of visibility was medium or poor (*p* = 0.003). The weather conditions during the crash were good in more than 91% for both the subjects who had head injuries and those who did not. The road surface was in good condition at the time of the crash for both those with head injuries (78.5%) and those without (79.6%). The occurrence of head injuries did not depend on the time of day, weather conditions, road conditions, and even less on the type of road on which the crash occurred (Table 3).

**Table 3:** Distribution of cases and controls according to factors related to the road and the environment

*Variables*	Head injuries (% or Mean ± sd)		p-value
	Yes (*n* = 242)	No (*n* = 484)	
Type of road			0.397
*National Inter-State Road*	18.2	13.8	
*Rural track*	6.2	5.2	
*National road*	21.5	22.1	
*Alley*	54.1	58.9	
Road condition			0.804
*Good*	78.5	79.6	
*Poor*	17.8	16.1	
*Under construction*	3.7	4.3	
Antagonist			0.006
*No antagonist*	19.8	12.4	
*Stationary vehicles or obstacles*	8.3	5.4	
*Moving vehicles*	64.9	77.1	
*Pedestrians, animals or moving objects*	7.0	5.2	
Weather conditions			0.775
*Good*	92.2	91.5	
*Bad*	7.8	8.5	
Visibility			0.003
*Good*	64.5	76.2	
*Acceptable*	14.4	11.0	
*Poor*	21.1	12.8	
Time of day			0.070
*Dusk*	14.9	15.1	
*Dawn*	6.2	6.2	
*Day*	45.9	54.5	
*Night*	33.0	24.2	

### Motorcycle rider history

The percentage of head trauma patients with a medical history was 11.2 and 19.8% for the cases and the controls, respectively (*p* = 0.003). The use of sleeping pills, stimulants or tobacco was infrequent among both the cases and the controls. For sleeping pills, this consumption was less than 3% in the two groups. The consumption of stimulants varied between 5.4% in the subjects with head injuries and 6.2% in the subjects without head injuries. Tobacco was consumed by 10.7% of the cases versus 12.0% of the controls. Consumption of alcoholic beverages was common (65.3% in the subjects with head injuries versus 67.8% in the subjects without head injuries). Driving experience was approximately 16 years in both groups. More than a third of the subjects had already been in a traffic crash before the current crash among cases and controls (39.3% versus 35.3%). There was no difference between the two groups regarding the consumption of the different substances, their years of driving experience or their history of traffic crashes (Table 4).

**Table 4:** Distribution of cases and controls according to history

*Variables*	Head injuries (% or Mean ± sd)		*p*-value
	Yes (*n* = 242)	No (*n* = 484)	
Medical history			0.003
*Yes*	11.2	19.8	
*No*	88.8	80.2	
History of traffic crashes			0.327
*Yes*	39.3	35.3	
*No*	60.7	64.7	
Sleeping pill consumption			1.000
*Yes*	2.9	2.9	
*No*	97.1	97.1	
Consumption of stimulants/doping substances			0.657
*Yes*	5.4	6.2	
*No*	94.6	93.8	
Consumption of alcoholic beverages			0.503
*Yes*	65.3	67.8	
*No*	34.7	32.2	
Tobacco consumption			0.623
*Yes*	10.7	12.0	
*No*	89.3	88.0	
*Motorcycle riding experience (years)*	15.7 (10.3)	16.8 (10.2)	0.177

### Factors associated with head injuries

In multivariate analysis, the factors associated with head injuries in two-wheel motorcycle riders in Benin were: wearing a helmet, driving while tired or drowsy, and a medical history. Considering the medical history and the notion of driving in a state of fatigue, subjects who did not wear helmets were at greater risk of head injuries than those who wore helmets (AOR = 3.8 (95% CI = 2.5–5. 7)). Individuals driving in a state of fatigue were 1.9 (95% CI = 1.2–3.1) times more likely to have head injuries than those who did not drive in a state of fatigue, taking into account helmet wearing and medical history. Adjusting for the other variables, subjects with no medical history were 2.0 (95% CI = 1.2–3.3) times more likely to have head injuries compared to those with a medical history (Table 5).

**Table 5:** Factors associated with head injuries in trauma patients, in univariate and multivariate analysis

Variables	OR (IC à 95%)	ORaj (IC à 95%)
**Helmet use**		
*Yes*	1	1
*No*	4.0 (2.7–6.0) ***	3.8 (2.5–5.7) ***
**Fatigue/drowsiness during the crash**		
*No*	1	1
*Yes*	1.9 (1.2–3.1) **	1.9 (1.2–3.1) **
**Medical history**		
*Yes*	1	1
*No*	2.0 (1.2–3.1) **	2.0 (1.2–3.3) **
**Sex**		
*Female*	1	
*Male*	1.8 (1.0–3.2) **	
**Telephone use during the crash**		
*No*	1	
*Yes*	4.1 (1.2–13.8) **	
**Antagonist**		
*Moving vehicles*	1	
*No antagonist*	1.9 (1.2–2.9) **	
*Stationary vehicles or obstacles*	1.8 (1.0–3.7) *	
*Pedestrians, animals or moving objects*	1.6 (0.8–3.1)	
**Time of day**		
*Day*	1	
*Dusk*	1.2 (0.7–1.9)	
*Dawn*	1.2 (0.6–2.3)	
*Night*	1.6 (1.1–2.3) **	
**Visibility**		
*Acceptable*	1	
*Good*	1.6 (1.00–2.5) *	
*Poor*	1.9 (1.3–2.9) **	

*p < 0.1; **p < 0.05; ***p < 0.001

## Discussion

In this study, the mean age of victims was 35.6(12.7) years for cases and 36.7(12.1) years for controls. This founding is in line with previous studies, which found that the age of subjects involved in road crashes ranged from 25 to 35 years old and up to 38 years old (Tumwesigye et al. 2016; Brown et al. 2011; Tidjani et al. 2018; Hode et al. 2017; Lam et al. 2019; Xiong et al. 2016; Chalya et al. 2014). As in our study, some previous studies did not found age associated with head injuries (Orsi et al. 2014; Lam et al. 2020; Walle et al. 2018; Kamulegeya et al. 2015). However, studies in Taiwan, China and Nigeria reported age as a factor associated with injury severity (Lam et al. 2019; Xiong et al. 2016) and or maxillofacial injuries (Oginni et al. 2006).

The majority of motorcycle trauma victims were men in this study. This findings were similar to most others studies, (Brown et al. 2011; Tidjani et al. 2018; Hode et al. 2017; Lam et al. 2019; Xiong et al. 2016; Allen et al. 2017; Chalya et al. 2012; Cavalcanti et al. 2014). The finding that there were not relations between gender and head injuries is in agreement with studies in Germany, Brazil and Taiwan (Orsi et al. 2014; da Nóbrega et al. 2017; Lam et al. 2020). In contrast to our results, gender was associated with the occurrence of head injuries among traumatized persons in Ethiopia (Walle et al. 2018). This difference could be explained by the fact that this Ethiopian study takes into account all causes of trauma and not only those related to road crashes.

There was no association between occurrence of head injuries and others sociodemographic and economic factors. These findings were in accordance with others studies. Previous studies did not found association between full time job and head/neck injuries in Taiwan (Lam et al. 2020) or employment status and facial trauma in Brazil (da Nóbrega et al. 2017). Kamulegeya et al. did not observe an association between the occurrence of head injuries and ethnicity (Kamulegeya et al. 2015) and a study in Ethiopia did not reported association between duration of having driving licence prior the accident and injury severity (Baru et al. 2019). In contrast, the prevalence of injuries were higher among single individuals compared to those married in Uganda and Brazil. These authors explain this difference by the risk behaviours of young subjects without family responsibility. (Tumwesigye et al. 2016; da Nóbrega et al. 2017).

The proportion of motorcycles trauma victims wearing a helmet is much higher in this study (90.3% in subjects without head injuries vs. 69.8% in those with head injuries) than that observed in 2013 and even in 2014 in Benin (Tidjani et al. 2018; Hode et al. 2017), in Ethiopia (Baru et al. 2019), in Uganda (Kamulegeya et al. 2015) and in Taiwan (Lin et al. 2003). Our study confirmed that in Benin, motorcycle riders traumatised in road crashes while wearing helmets were less likely to have head injuries. Several other authors have come to similar conclusions (Brown et al. 2011; Kamulegeya et al. 2015; Bachani et al. 2017). For instance, using hospital data from four hospitals in Kenya, Bachani et al. noted that subjects wearing helmets had a lower risk of head trauma (OR = 0.478) than those who did not (Bachani et al. 2017). The same result was found by Kamulegeya et al. in Kampala among motorcycle taxi drivers. Drivers who did not wear helmets were 2.3 times more likely to have head injuries than those who did (*p* = 0.004) (Kamulegeya et al. 2015). Singleton found this significant association, regardless of the type of head injury (uncomplicated concussion, brain contusion, intracranial haemorrhage and skull fracture) (Singleton 2017). Brown et al. and Phillips et al. observed in the USA that different lesions with intracranial trauma were significantly more present in subjects not wearing a helmet than in those who did (Brown et al. 2011; Phillips et al. 2017). For other authors, there was a relationship between injury severity (AIS) and helmet use (Baru et al. 2019; Lam et al. 2019; Xiong et al. 2016; Chalya et al. 2014), and not wearing a helmet would increase the risk of sustaining road traffic injuries (Tumwesigye et al. 2016) or the risk of death from road crashes among motorcyclists (Chang et al. 2016; Boniface et al. 2016). According to Lam et al., head injuries are more likely to be associated with the use of non-standard helmets (Lam et al. 2020). Others authors founded relations between maxillofacial injuries and helmet use (Usha et al. 2014). Contrary to most studies, Orsi et al. did not find this association in their multivariate analysis (Orsi et al. 2014).

Bougard et al., in a literature review reported that fatigue and sleepiness might affect riding capabilities, increasing the risks of being involved in an accident and might cause more serious damage because of the lack of reaction of the rider(s) involved (Bougard et al. 2016). Indeed, in Vietnam, 16% of the motorcycle taxi driver reported fatigue-related crash involvement (Truong et al. 2020). The same trend was observed in our study among trauma patients; 16.9% of trauma patients with head injuries and 9.3% of those without head injuries were fatigue/drowsiness during the crash. In our adjusted model, fatigue was associated with the occurrence of head injuries. This issue is comparable with the findings in Taiwan were fatigue riding was a risk factor for severe injury (OR: 1.85, 95% CI: 1.07–3.20) (Lam et al. 2019).

Use mobile phone while riding was not a factor associated with head injuries in our multivariate model. In contrast, previous study in Taiwan founded that motorcyclists using the mobile phone at the time of crash were twice as likely to suffer head injuries (Lam et al. 2020).

Factors related to road and environment conditions were not found to be associated with occurrence of head injuries in our study. This was in agreement with some previous studies. Thus, association between facial trauma and period of day was not founded in multivariate analysis in Brazil (da Nóbrega et al. 2017), antagonist was not liked to severe injury in Taiwan (Lam et al. 2020) and the weather conditions and road conditions were not factors associated with head injuries in Germany (Orsi et al. 2014). In contrast, some authors found, in their studies, association with time of day (Lin et al. 2003; Baru et al. 2019; Lam et al. 2020; Bachani et al. 2017), visibility (Xiong et al. 2016) and antagonist in the crash (Orsi et al. 2014; Kamulegeya et al. 2015). The influence of antagonist and/or the mechanism of the crash was also identified by some previous studies in factors associated with severe injury in road traffic injuries (Baru et al. 2019; Lam et al. 2019; Xiong et al. 2016) and as factor associated to maxillofacial injuries (Oginni et al. 2009; Oginni et al. 2006).

We found in our study that subjects with a history of disease had significantly less head trauma than those with no history. Few studies have looked for this relationship. A study in Taiwan found no links between different medical histories except for anemia. However, contrary to our results, subjects suffering from this medical history were more at risk of head trauma (Lam et al. 2020). In our study, this observation could be explained by the fact that these subjects, aware of their fragile state of health pay more attention while driving.

In our study, individuals who consume alcohol are not more likely to have head injury. Previous studies have found an association between alcohol intoxication and head injuries particularly trauma brain injuries (Lam et al. 2020; Weil et al. 2018) or injury severity (Baru et al. 2019). The relationship with acute intoxication could not be explored in our study.

Sleeping pill, stimulants/doping and tobacco consumption did not increase the risk of head injuries in this study. Lam et al. did not also found association with medication before riding (Lam et al. 2020).

In our study, we noted that almost a third of trauma patients enrolled had previously been in a traffic crash but there were not association with head injuries. This significant proportion should make the actors involved in road safety reconsider education and communication strategies for a change in user behavior.

### Limitations of the study

The data was collected in five hospitals that are not necessarily representative of the country. Many data were collected from the casualties, so there is a possibility of information bias related to the fact that all of these variables were entered based on the declarations of the targets. The retrospective nature of some data and the fact that some questions are memory-dependant can also cause recall bias. In addition, the reluctance of some people to give their personal information and the inability of some patients who are severely disabled or died, resulted in some missing data, especially regarding conditions of the crash and certain behavioural variables such as helmet wearing, consumption of alcohol or psychoactive substances before driving, respect for signs and priorities, excess speed, etc. Finally, because of hospital data collection, it was not possible to collect data on speeding, blood alcohol level before the accident. This lack of information did not allow certain factors to be included into the analysis.

## Conclusion

This study showed that not wearing a helmet is one of the main risk factors for the occurrence of head injuries in motorcycle riders in a road crash along with other behavioural factors, such as driving while fatigued, or non-behavioural factors, such as medical history.

In view of these results, helmet wearing should continue to be enforced in Benin and extended to motorcycle passengers. Motorcyclists should be educated to avoid riding in a state of fatigue.

## Data Availability

The data and materials of this study will be available from the main author, but also from the ReMPARt project. To have access to these data, contact the main author.

## Abbreviations

WHO: World Health Organization
ReMPARt: Multidisciplinary Research in the Prevention of Road Accidents
